# Deformable microparticles for shuttling nanoparticles to the vascular wall

**DOI:** 10.1126/sciadv.abe0143

**Published:** 2021-04-21

**Authors:** Margaret B. Fish, Alison L. Banka, Margaret Braunreuther, Catherine A. Fromen, William J. Kelley, Jonathan Lee, Reheman Adili, Michael Holinstat, Omolola Eniola-Adefeso

**Affiliations:** 1 Department of Chemical Engineering, University of Michigan, Ann Arbor, MI 48109, USA.; 2 Department of Pharmacology, University of Michigan, Ann Arbor, MI 48109, USA.; 3 Department of Cardiovascular Medicine, Samuel and Jean Frankel Cardiovascular Center, University of Michigan, Ann Arbor, MI 48109, USA.; 4 Department of Biomedical Engineering, University of Michigan, Ann Arbor, MI 48109, USA.; 5 Macromolecular Science and Engineering Program, University of Michigan, Ann Arbor, MI 48109, USA.

## Abstract

Vascular-targeted drug carriers must localize to the wall (i.e., marginate) and adhere to a diseased endothelium to achieve clinical utility. The particle size has been reported as a critical physical property prescribing particle margination in vitro and in vivo blood flows. Different transport process steps yield conflicting requirements—microparticles are optimal for margination, but nanoparticles are better for intracellular or tissue delivery. Here, we evaluate deformable hydrogel microparticles as carriers for transporting nanoparticles to a diseased vascular wall. Depending on microparticle modulus, nanoparticle-loaded poly(ethylene glycol)–based hydrogel microparticles delivered significantly more 50-nm nanoparticles to the vessel wall than freely injected nanoparticles alone, resulting in >3000% delivery increase. This work demonstrates the benefit of optimizing microparticles’ efficient margination to enhance nanocarriers’ transport to the vascular wall.

## Teaser

Hydrogel microparticle carriers delivered more nanoparticles to the vascular wall in vivo than ones freely injected.

## INTRODUCTION

Vascular-targeted drug carriers (VTCs) are typically polymeric particles engineered to adhere to and accumulate at sites of disease via markers on the vascular wall, producing localized delivery of drugs. Several publications have established that drug carriers’ physical properties determine their circulation time, biodistribution, vascular wall adhesion, and immune interactions (*1*–*4*). For VTCs, efficient vascular wall localization, i.e., successful margination from bulk blood flow, is a critical prerequisite for adhesion and the eventual release of their drug payload to the diseased endothelium or tissue.

Biocompatible, biodegradable nanoparticles (NPs) are appealing drug carrier candidates, with NPs of 20 to 80 nm in diameter able to avoid immune recognition and clearance (*5*, *6*). The high surface area–to–volume ratio of NPs also allows rapid drug release from the carrier (*7*), while their physical size facilitates transcytosis into adjacent tissues (*8*, *9*). However, recent works reveal that less than 1% of injected NP doses reach the intended site (*10*, *11*), which may be linked to the recently highlighted diminished ability of NPs to marginate, i.e., localize to the vascular wall (*12*–*14*). Although 2- to 3-μm-diameter microparticles (MPs) are reported to exhibit high margination and appear optimal as VTCs (*13*, *15*), these microcarriers still face challenges, including the potential for short circulation times, dangerous capillary occlusions for rigid MPs, and the lack of intercellular delivery benefits unlike NPs (*16*). Furthermore, the ability of the targeting ligands grafted on carrier surfaces to bind overexpressed receptors on the diseased vascular wall has been debated in the literature; ligands are often proteins, which are thought to decrease the circulation time and half-lives of particles significantly (*17*, *18*). Some use this fact alone as a rationale to abandon the targeted particle strategy, focusing instead on extending the circulation time for passive accumulation. Thus, researchers have recently established particle Young’s modulus or the tensile stiffness of a material as a critical physical property for VTCs. Lower modulus decreases nonspecific particle entrapment and slows phagocytosis by immune cells (*19*, *20*). Conversely, if particles rapidly bind to the target site, the circulation half-life may not be the only defining particle characteristic. To date, these competing paradigms have yet to be directly tested experimentally.

This study examines the possibility of loading NPs into vascular-targeted, deformable MPs to overcome the previously highlighted transport limitation faced by NPs. We find that size and MP modulus are the controlling variables of overall particle delivery to the vascular wall, with targeting moieties providing a necessary component to sustain particle accumulation. We show that NP-loaded hydrogel MPs effectively deliver more NPs to the vascular wall than free NPs. Overall, this work offers an avenue to increase the clinical utility for NP VTCs, with applications in many common diseases.

## RESULTS

### Fabrication and material characterization of NP-loaded hydrogel MPs

**Fig. 1:**
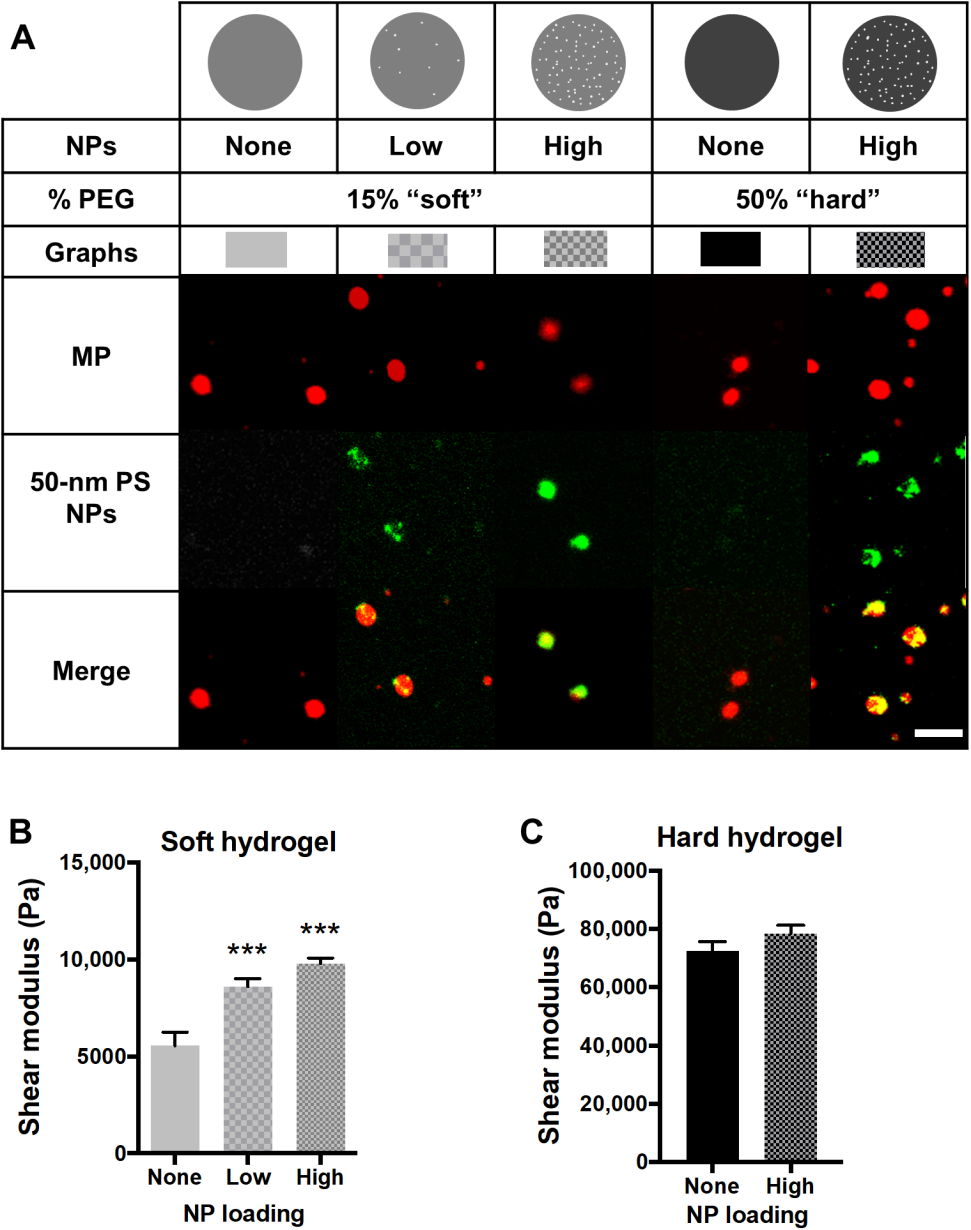
Material properties of NP-loaded hydrogel MPs. (**A**) Schematic and representative confocal microscopy fluorescence images of hydrogel MPs evaluated, having varied modulus and NP loading. Red is MP hydrogel, green is 50-nm PS NPs, and the two are overlaid to show colocalization of NPs and hydrogel MPs. Scale bar, 5 μm. Swollen shear moduli for (**B**) 15% PEG and (**C**) 50% PEG hydrogels showing the influence of adding NPs to bulk material rheometry. Statistical analyses were performed using one-way analysis of variance (ANOVA) with Fisher’s least significant difference (LSD) test, where (***) indicates *P* < 0.001 in comparison to the nonloaded hydrogels. *N* = 3. Error bars plot SE.

To overcome the pervasive transport limitations of NPs to the vascular wall, we fitted our previously described (*4*) deformable hydrogel MP carriers with polymeric NPs as the cargo. Reaction conditions for the unloaded poly(ethylene glycol) (PEG) hydrogels, physical properties, and representative scanning electron microscopy images of unloaded and loaded hydrogels are detailed in fig. S1. We investigated how different emulsion fabrication variables affected the loading of fluorescent, 50-nm polystyrene (PS) NPs into 2-μm-diameter spherical hydrogel MPs. Although we could achieve poly(lactide-*co*-glycolide) (PLGA) NP-loaded MPs (fig. S2), we opted to focus this work on PS NPs because of their uniform size distribution, allowing consistency of NP load across different MP formulations. Thus, we focus on the deformable MPs’ capacity to deliver NPs to the vascular wall. We evaluated the importance of PEG diacrylate (PEGDA) precursor’s molecular weight and composition in the final NP loading into hydrogel MPs (fig. S3), and we developed optimized formulations for two different % solids of the 700-Da molecular weight monomer—15 and 50%. Our series of MPs include “soft” 15% PEG particles with a low and high NP load and a “hard” 50% PEG particle with one NP loading density. The schematic and fluorescent images of these particles are shown in the top panel of Fig. 1A. On the basis of flow cytometry analysis (fig. S3), we achieved an average of 3 and 25 PS NPs per soft MPs for the low and high load, respectively. The hard NPs contained 16 NPs per MPs. We show these NP-loading rates to be consistent over multiple hydrogel batches (fig. S1). Calculated hydrogel pore sizes are 3.5 and 0.3 nm for the 15 and 50% PEG conditions, respectively (*21*); thus, we do not expect any diffusion of the NPs out of the MPs.

Given that vascular-targeted carrier modulus has been shown to affect particle adhesion efficiency (*19*, *20*), we tested how the physical loading of rigid PS NPs, with an elastic modulus of about 2 GPa (*22*), affected the bulk modulus of the hydrogels. In Fig. 1B, we show that the shear modulus of the soft hydrogels increased from 5600 ± 700 Pa (unloaded hydrogel) to 8600 ± 420 Pa for the low and 9800 ± 290 Pa for the high NP loading, as measured on swollen bulk hydrogels via an AR-G2 rheometer (*4*). The loading of 50-nm PS NPs into hard MPs did not significantly increase the bulk shear modulus (72,500 ± 300 to 78,400 ± 280 Pa), as shown in Fig. 1C. Overall, despite the significant impact of NP loading on the rigidity of the soft hydrogel, these hydrogels remain considerably more deformable than the hard hydrogels.

### Hydrogel MPs outperform free NPs in an experimental blood vessel model

**Fig. 2:**
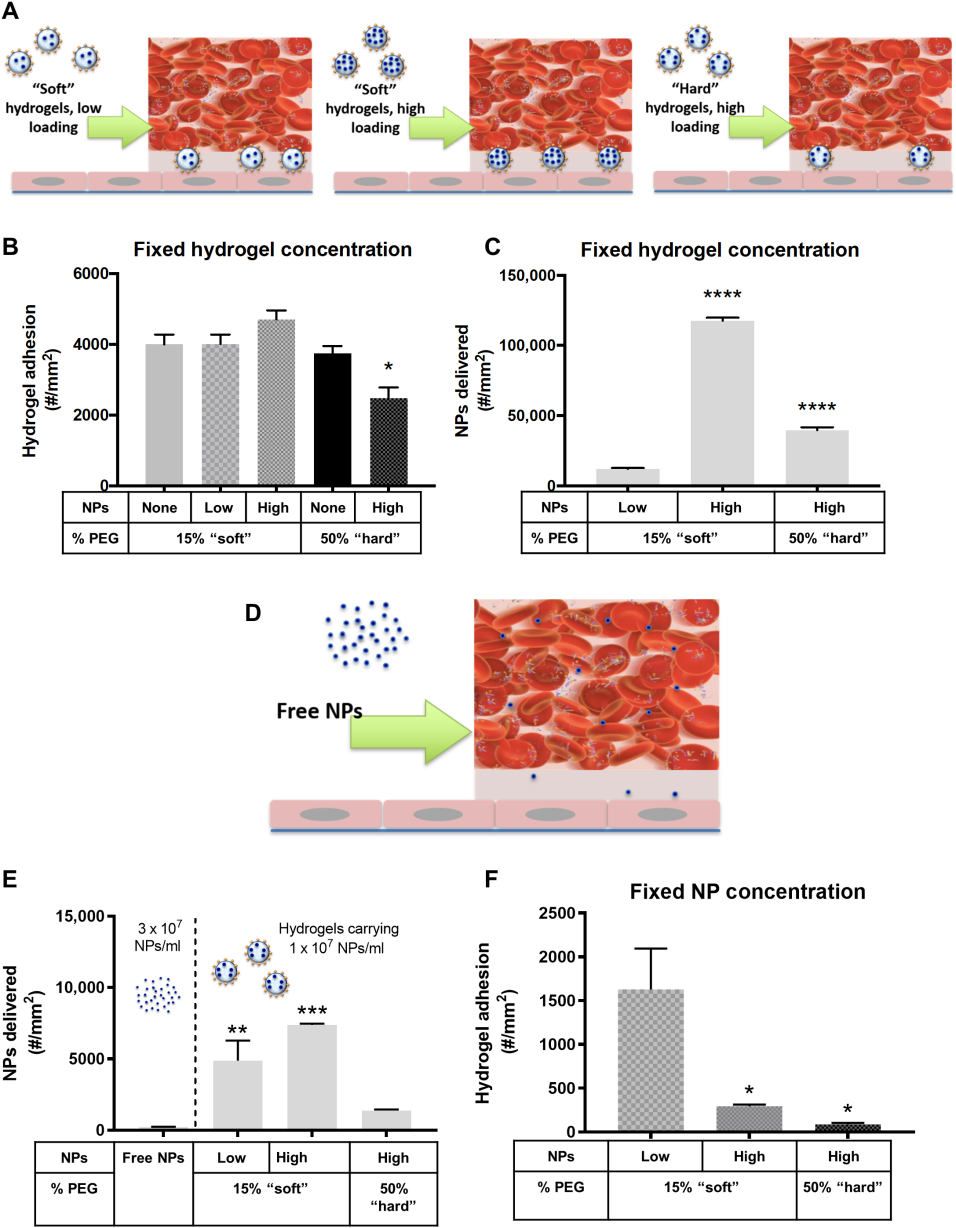
Adhesion of NP-loaded hydrogel MPs to an inflamed HUVEC monolayer at 200 s^−1^ WSR. (**A**) Schematic detailing “fixed MP concentration” in vitro flow experiments. Quantified (**B**) adhesion for anti–ICAM-1–coated hydrogel MPs dosed in blood at a fixed MP concentration and scaled to (**C**) the corresponding number of NPs delivered by the adherent hydrogel MPs in (B). (**D**) Schematic of the free NP in vitro flow experiments. (**E**) Number of NPs delivered to the vascular wall by free anti–ICAM-1–coated PS NPs dosed at 3 × 10^7^ NPs/ml or based on (**F**) the adhesion of hydrogel MPs dosed in blood to carry a fixed three times lower NP cargo of 1 × 10^7^ NPs/ml. For all, adhesion was quantified after 5 min of laminar blood flow over an IL-1β–activated HUVEC monolayer. *N* ≥ 3 human blood donors per particle condition. Statistical analysis of adherent density was performed using one-way ANOVA with Fisher’s LSD test, where (*) indicates *P* < 0.05, (**) indicates *P* < 0.01, (***) indicates *P* < 0.001, and (****) indicates *P* < 0.0001 versus the first bar in each plot. Error bars plot SE.

Next, we evaluated the ability of our NP-loaded hydrogel MPs to bind to an activated human umbilical vein endothelial cell (HUVEC) monolayer from human blood flow in a parallel plate flow chamber (PPFC) with a channel height of 127 μm (*4*). Using the PPFC assay, we can quantify the number of MP and NPs trafficked to the wall. Each MP type was conjugated with 5000 anti–ICAM-1 (intercellular adhesion molecule–1)/μm^2^, which was confirmed via flow cytometry, and all experiments were at a low or high wall shear rate (WSR) of 200 or 1000 s^−1^, respectively. First, we evaluated MPs’ adhesion with a fixed MP concentration (number) present in the blood as depicted in Fig. 2A. Figure 2B shows the targeted MP adhesion density after 5 min of blood flow at 200 s^−1^ for the condition of a fixed hydrogel MP concentration in blood. The soft 15% PEG particles adhered equivalently independent of NP loading (unloaded, low, and high), suggesting that the change in modulus shown in Fig. 1 had minimal impact on MP adhesion under low shear, as previously described (*4*). In contrast, the hard 50% PEG MPs adhered less than all soft conditions and relative to the unloaded counterpart. We found a similar adhesion pattern for hydrogel MPs loaded with PLGA NPs, indicating the translational nature of the results for human use (fig. S2).

To determine the number of NPs transported to the vessel wall for the fixed MP number dosing scheme, we compared the loaded hydrogel MPs to free NPs on a plate reader to determine the average number of NPs loaded per hydrogel MP. We translated the results from Fig. 2B to the number of 50-nm NPs delivered to the wall. Given the similar binding of soft MPs with a fixed MP concentration in blood, those with higher NP loading delivered a significantly higher NP payload to the wall (Fig. 2C).

To determine the efficiency of MPs for transporting NPs to the vascular wall, we compared the amount of NPs delivered by the worst-performing hydrogel MPs from Fig. 2C, i.e., the low load, soft MPs, to the amount of “free” NPs bound at a fixed number of NPs in blood. Therefore, we conducted assays to evaluate NP adhesion to wall from blood flow when free anti–ICAM-1–coated NPs were instead used at 3 × 10^7^ NPs/ml of blood (Fig. 2D), corresponding to the NP cargo carried by the low soft MPs in Fig. 2B. As shown in Fig. 2E, on average, ~217 NPs/mm^2^ were bound to the vessel wall in assays with free NPs in blood. Comparatively, as shown in Fig. 2C, the low load, soft MPs delivered ~12,000 NPs/mm^2^ to the wall despite the same blood NP concentration, representing 5450% more NPs at vessel wall versus free NPs.

Next, we evaluated the number of NPs delivered to the vessel wall by all hydrogel MP types dosed in the blood at concentrations such that they all carry a fixed NP amount of 1 × 10^7^ NPs/ml. That is, more hydrogel MPs/ml of blood were used for MPs carrying a low NP load, leading to a 3.3 × 10^6^/ml concentration for the 15% PEG low loading, 4 × 10^5^/ml for the 15% PEG high loading, and 6.25 × 10^5^/ml for the 50% PEG high loading. We reduced the hydrogel NP cargo to 1 × 10^7^ NPs/ml, i.e., one-third of the NP amount for the free NPs assays, as we were curious to see whether the lower number of hydrogels in blood would equalize the playing field with free NPs. As expected (based on results in Fig. 2B), the 15% PEG MPs with low NP loading adhere significantly more than other MP types due to their higher bloodstream concentration (Fig. 2F). Notably, the low and high loading soft MPs delivered 2150 and 3300% more PS NPs to the vessel wall than free NPs, respectively, despite the three times more free NPs in blood than the NP cargo in MPs. The hard MPs also delivered 540% more NPs to the wall than the free NPs, though not significant.

**Fig. 3:**
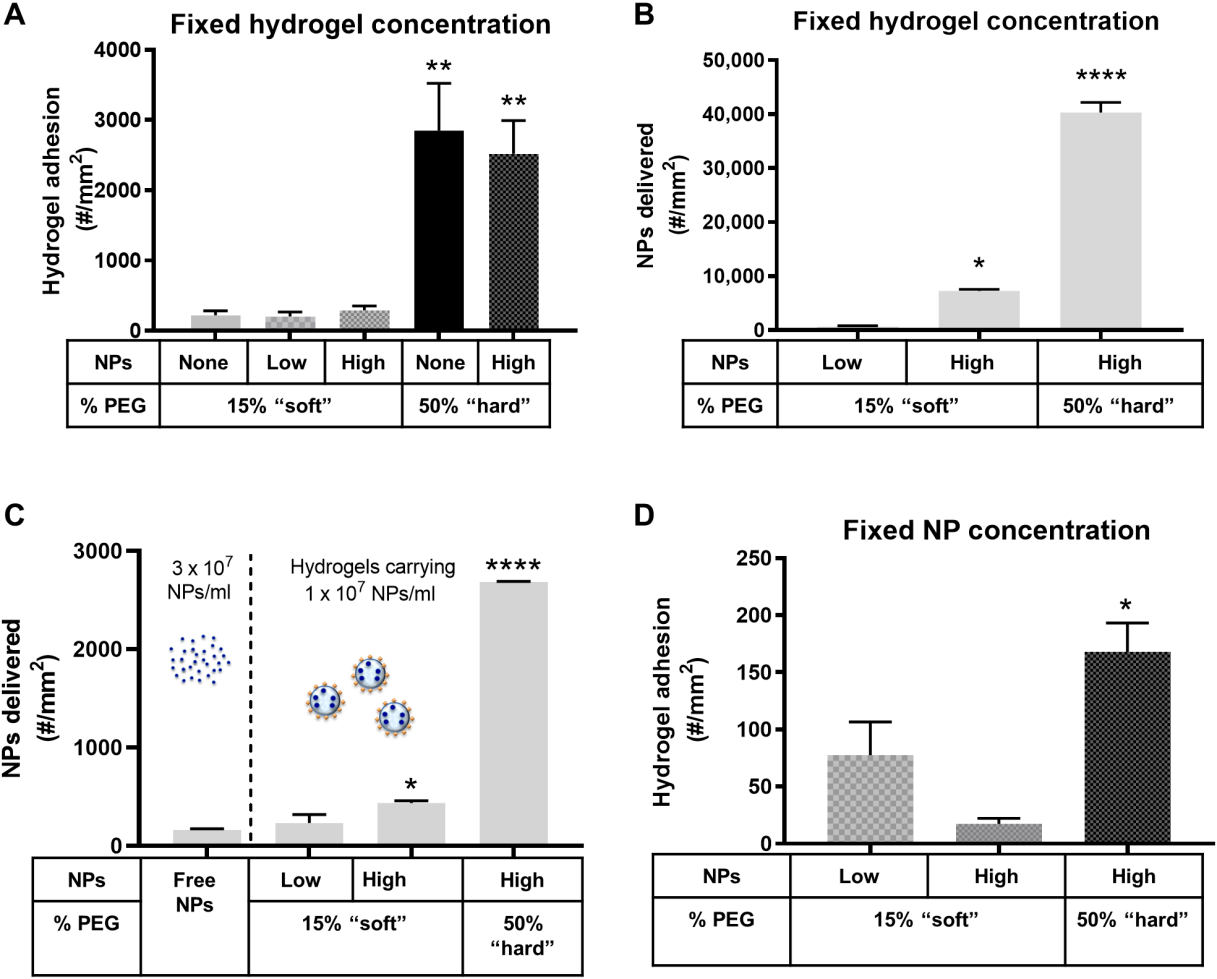
Adhesion of NP-loaded hydrogel MPs to an inflamed HUVEC monolayer at 1000 s^−1^ WSR. Quantified (**A**) adhesion for hydrogel MPs dosed in blood at a fixed MP concentration and scaled to (**B**) the corresponding number of NPs delivered by the adherent hydrogel MPs in (A). (**C**) Number of NPs delivered to the vascular wall by free anti–ICAM-1–coated NPs dosed at 3 × 10^7^ NPs/ml or based on (**D**) the adhesion of hydrogel MPs dosed in blood to carry a fixed three times lower NP cargo of 1 × 10^7^ NPs/ml. For all, adhesion was quantified after 5 min of laminar blood flow over an IL-1β–activated HUVEC monolayer. *N* ≥ 3 human blood donors per particle condition. Statistical analysis of adherent density was performed using one-way ANOVA with Fisher’s LSD test, where (*) indicates *P* < 0.05, (**) indicates *P* < 0.01, (***) indicates *P* < 0.001, and (****) indicates *P* < 0.0001 versus the first bar in each plot. Error bars represent SE.

We also evaluated particle adhesion at a high WSR of 1000 s^−1^. At a fixed hydrogel MP concentration in the high shear flow, the hard 50% PEG particles adhered significantly more than the soft 15% PEG particles (Fig. 3A). This hydrogel MP adhesion pattern is consistent with our previous finding that stiffer hydrogel MPs were more efficient at binding in high shear flow in vitro (*4*), which we show is not due to a change in MP localization pattern. Instead, this adhesion pattern is driven by deformability-associated differences in the impact of near-wall collisions with white blood cells (*4*). The difference in modulus produced by the loading of NPs within each of the soft and hard particle groups again did not affect adhesion significantly. We translated these hydrogel MP adhesion results to the NP amount at the vessel wall (Fig. 3B). We find that the hard 50% PEG particles delivered the most NPs, consistent with their significantly higher adhesion density under high shear.

Again, the density of NPs delivered to the wall by the worst-performing hydrogel carrier, i.e., the soft, low load MPs, of 615 NPs/mm^2^ (Fig. 3B) was 280% more than the ~160 NPs/mm^2^ adhesion density achieved with free NPs (Fig. 3C). Similar to the low shear assays, we dosed all MPs to carry a three times lower NP load (1 × 10^7^ NPs/ml) by altering the number of hydrogel MPs per milliliter in the flow assay and translated the achieved adhesion to the NPs delivered to the vessel wall (Fig. 3C). The corresponding MP adhesion data are shown in Fig. 3D. Similar to the low shear assays, every NP-loaded hydrogel MP condition delivered more NPs to the vessel wall than free anti–ICAM-1–conjugated NPs perfused at three times the concentration of NP load carried by the MPs at the high WSR of 1000 s^−1^. We find that the hard MPs delivered significantly more NPs to the vessel wall than all other MP conditions tested due to their substantially higher adhesion efficiency in high shear flow (see Fig. 3A). Quantitatively, in the order of Fig. 3C, the hydrogel MPs delivered 43, 170, and 1550% increases in the number of NPs reaching the wall versus free NPs (3 × 10^7^ NPs/ml).

### Low margination of free NPs in blood flow accounts for poor vessel wall binding

We conducted several control experiments to confirm that the discrepancy between NPs delivered to the vascular wall by MPs versus free NPs is not due to free NPs binding to blood cells or being phagocytosed by blood leukocytes. To start, we conducted a flow cytometry analysis of the blood samples collected after flow assays and found an insignificant number of leukocytes that were bound by NPs (fig. S4). We also found that a very minimal number of blood cells bind NPs in static assays where free NPs were incubated in blood for up to 10 min at the same concentration as used in the flow adhesion assays (fig. S5). To demonstrate that 50-nm NPs bound to blood cells can be detected, we evaluated the binding of 1 × 10^11^/ml NPs conjugated with anti-CD45. These targeted NPs are expected to bind leukocytes in whole blood relative to unconjugated NPs (negative control) in static assays. Like the free NPs in flow assays, unconjugated PS NPs did not bind to blood cells significantly even up to an hour (fig. S6). Conversely, we could detect moderate to high anti-CD45 NP binding to leukocytes (fig. S6). Last, we found that a minimal number of blood leukocytes phagocytosed 50-nm NPs in static assays for up to 1 hour (fig. S7). As such, we conclude that the low NP adhesion to inflamed HUVEC delivered by free NPs in blood flow assays is due to their failure to localize to the vessel wall and not due to either clearance via phagocytosis or nonspecific binding to blood cells.

## Hydrogel MPs deliver significantly more NPs to the vessel wall than free NPs in vivo

To assess the in vitro results’ clinical translation, we used intravital microscopy to evaluate NP-loaded MPs’ adhesion in direct comparison to free 50-nm NPs in vivo in mesentery veins in mice. We down-selected the particle types to the 15% PEG MPs that were efficient at binding to the wall at low WSR (Fig. 2), as mesenteric veins are expected to have a low WSR in vivo (*23*). We dosed a fixed number of NPs, meaning a variable number of hydrogel MPs, all with a surface targeting ligand site density of 30,000 anti–P-selectin/μm^2^ to bind P-selectin induced by the topical application of tumor necrosis factor–α (TNF-α) on the mesentery vessels (*24*, *25*). The ligand site density was confirmed via flow cytometry. We chose to evaluate the particles in the mesentery with acute inflammation because of the ability to visualize particle adhesion live via intravital microscopy.

**Fig. 4:**
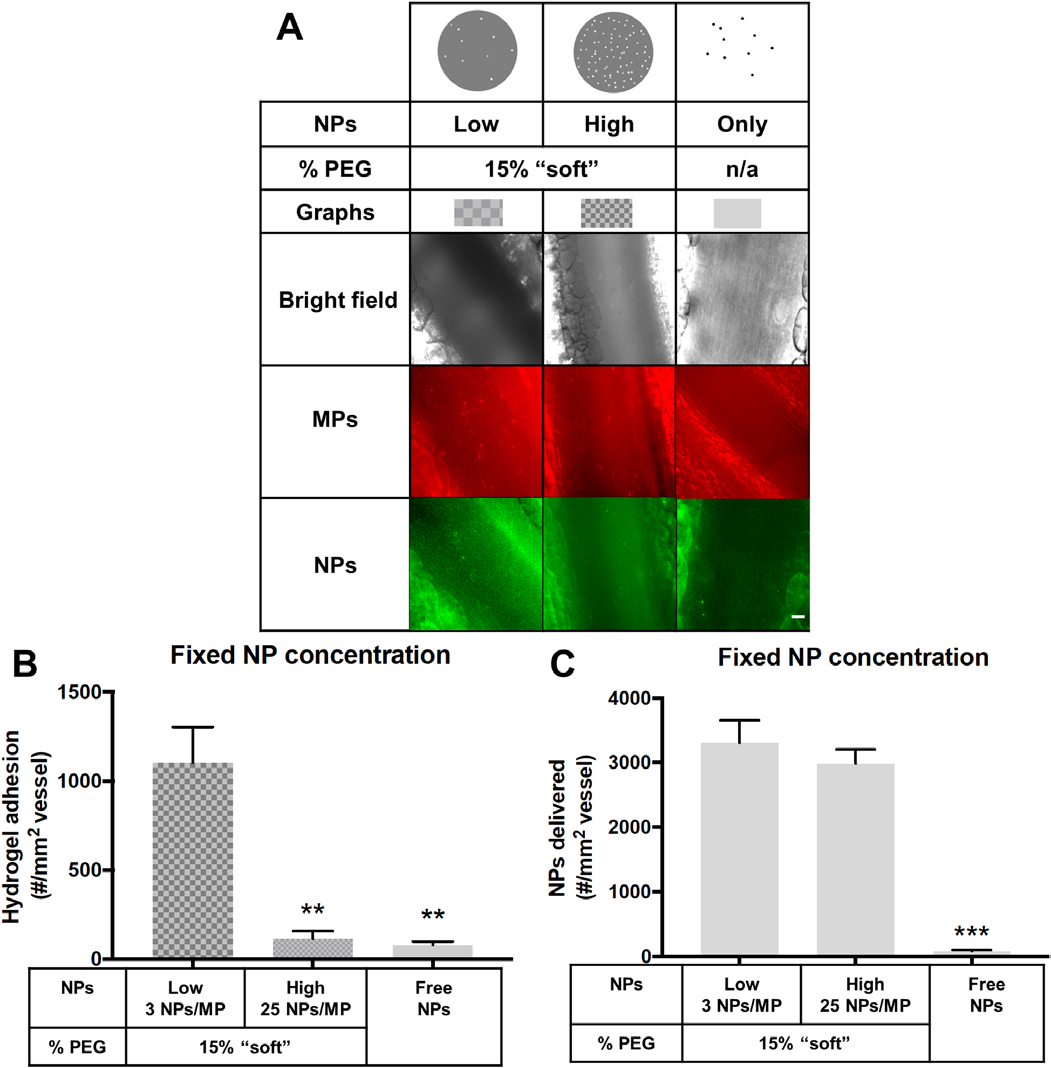
Delivery of NPs to an inflamed mesentery endothelium as a function of loading into hydrogel MPs. (**A**) Representative bright-field and fluorescence images of particle adhesion to inflamed mesentery. n/a, not applicable. (**B**) Quantified adhesion density of three different particle conditions, 15% PEG, low loading hydrogel MPs, 15% PEG, high loading hydrogel MPs, and free NPs. Particles were dosed by equivalent NP payload. (**C**) Data scaled to the number of NPs delivered by adherent hydrogel MPs to show the efficiency of NP delivery by each VTC system. *N* = 3 mice per group, and statistical analysis was performed using one-way ANOVA with Fisher’s LSD test, where (**) indicates *P* < 0.01 and (***) indicates *P* < 0.001 compared to the low NP–loaded 15% PEG. Error bars plot SE. Scale bar, 50 μm.

Figure 4A shows representative images of each particle type bound to a TNF-α–activated mesentery vein after 5 min of circulation time, with hydrogel MP in red, NP in green, and merged to observe the overlap. Figure 4B shows the vascular wall adhesion of hydrogel MPs/mm^2^ after 5 min of intravenous injection. Again, the soft MPs with a low NP load produced the best hydrogel MP adhesion because of more particles injected than the other hydrogel MP type. In Fig. 4C, we translated these results to the number of NPs delivered and found that in vivo, the loading density of NPs does not significantly change the number of NPs trafficked to the vessel wall within the soft particle type. However, free 50-nm PS NPs still resulted in substantially less adhesion versus high and low loading of NPs into the 15% PEG particles. Specifically, the two hydrogel conditions resulted in, at a minimum, a 3630% increase in the number of NPs that arrive on the vessel wall. Overall, hydrogel MPs were significantly more efficient at delivering 50-nm PS NPs to an inflamed mesentery in vivo versus free NPs, regardless of the number of NPs loaded. Representative images of the inflamed mesentery before NP injection, as well as images of clearly adherent NPs to the mesentery, are available in fig. S8.

### Sustained adhesion over time: Larger, softer particles are best at maintaining adhesion

**Fig. 5:**
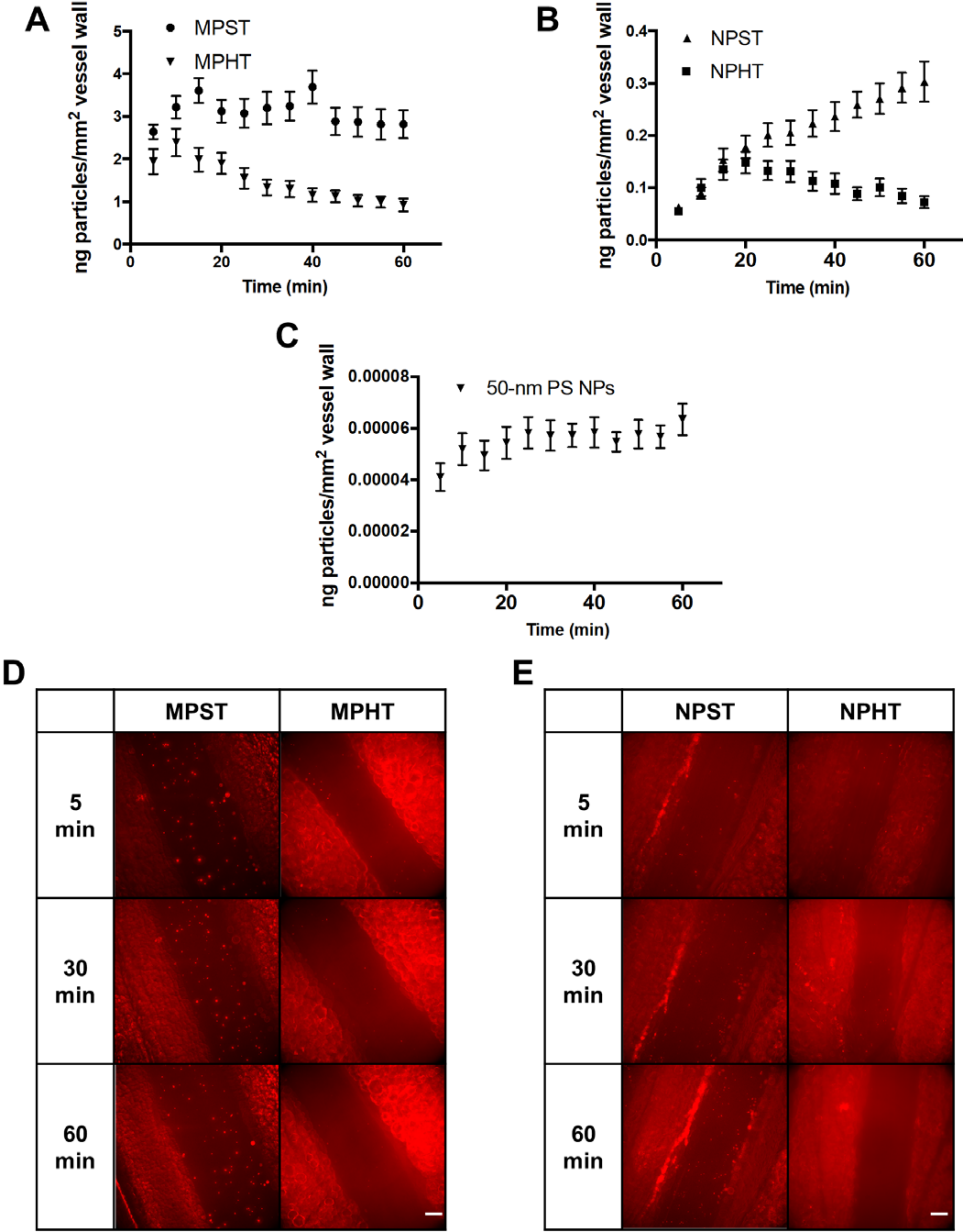
Intravital microscopy analysis of MP and NP adhesion over time. The adhesion of particles on an inflamed mesentery vein for (**A**) 2-μm PEG, (**B**) 500-nm PEG, and (**C**) 50-nm PS particles over an hour. Error bar represents SE for *N* = 3. Representative images of mesentery adhesion of (**D**) 2-μm and (**E**) 500-nm PEG NPs at 5, 30, and 60 min after particle injection. MPST, targeted soft MP; MPHT, targeted hard MP; NPST, targeted soft NP; NPHT, targeted hard NP. Scale bar, 50 μm.

NPs have consistently been shown to have longer circulation times than micrometer-sized particles (*5*); thus, it is possible that, over time, the 50-nm PS particles can outperform MPs. To establish the potential trade-off between circulation time and targeted adhesion, we evaluated a series of intravenously dosed targeted PEG-based hydrogel particles in healthy mice over time. We sought to assess targeted particles’ binding duration; three deformable particle types were investigated and compared directly to the 50-nm PS particles. The soft hydrogel MPs of 2 μm diameter from above (15% PEG) were designated as MPST, and 500-nm-diameter particles of 15% PEG and 50% PEG composition were identified as NPST and NPHT, respectively, where “S” or “H” refers to either soft or hard modulus, and T signifies that these are antibody targeted. We chose to evaluate the 500-nm deformable PEG particles to determine the impact of size without differential surface chemistry. All particle types were conjugated with the same targeting ligand density of 30,000 anti–P-selectin/μm^2^. The same mass of each particle type was injected via a tail vein catheter so that the results shown in Fig. 5 are represented by mass. We captured particle adhesion in five distinct locations of the mesentery veins every 5 min for an hour.

Figure 5A shows the MPS adhesion over an hour, displayed as the mass of particle adherent per vessel area. The adhesion density increased with time in the first 10 min after particle injection. By 20 min, the adhesion density of the MPST was steady, which was maintained for up to an hour. Figure 5B shows the vessel wall adhesion for 500-nm NPs of both moduli. Two apparent trends emerged, one by size and one by modulus. First, the MPST bound significantly better than both the NPST and NPHT. The hydrogel NPs never matched or surpassed hydrogel MPs in targeted adhesion efficiency in this hour-long time frame (see fig. S9 for a plot of both particles on the same scale). The NPHT began to detach by 20 min of the particle injection, dropping to about half the maximal adhesion achieved by 1 hour. Soft 500-nm NP adhesion increased through the hour. Therefore, the superiority of the soft NPs versus their hard counterparts increased over the hour time window tested despite the uniform ligand density used on the particle surfaces.

To determine whether the hard 500-nm PEG particles’ detachment was due to their modulus or a combination of size and modulus, we evaluated the in vivo adhesion of 2-μm MPs with a 100% PEG composition, designated as MPHT in Fig. 5A. Similar to the NPHT relative to NPST, the targeted adhesion of the MPHT particles decreased over time relative to MPST, starting at the 15-min time point, despite the same targeting ligand density. This result suggests that the detachment observed with PEG particles is a function of the particles’ stiffness. Last, Fig. 5C shows 50-nm PS particles’ adhesion having the same anti–P-selectin density as the PEG particles. The adhesion of 50-nm NPs increased 54% from the initial value at the 5-min time point but leveled off and stopped increasing by 25 min. The binding of 50-nm NPs is nominal compared to that of all hydrogel MPs, demonstrated in the difference in *y*-axis values. Figure 5 (D and E) shows representative images of adhesion to an inflamed mesenteric vein 5, 30, and 60 min after particle injection for 2-μm and 500-nm hydrogel particles.

### Accumulation of PEG particles in the inflamed murine lungs

Given the deformable MPs’ notably better performance relative to NPs over an hour, we set to evaluate a longer targeting window with an acute lung injury model; intravital microscopy was constrained to an hour time window because of animal protocol limits. Lipopolysaccharide (LPS) was instilled into the mouse airways to induce a local inflammatory event. An equivalent mass of particles (30 mg of particles per kilogram of mouse, ~0.6 mg of particles) was injected via the tail vein 1 hour after LPS. We then quantified accumulation in the targeted organ of interest, the lung. Particles were either targeted or untargeted; anti–ICAM-1 was used as the targeting ligand for these experiments, as the lung endothelium highly up-regulates ICAM-1 through 48 hours after LPS instillation (*26*, *27*).

**Fig. 6:**
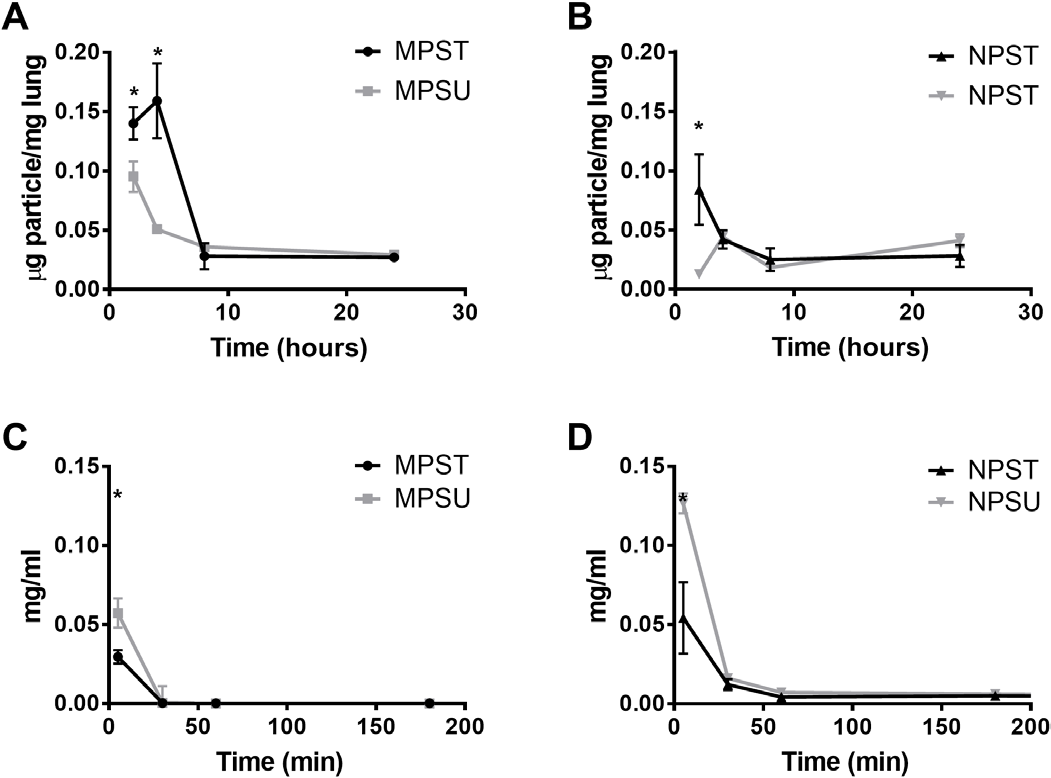
Behavior of targeted hydrogel particles in mice with acute lung injury. Accumulation of PEG-based (**A**) 2-μm MPs and (**B**) 500-nm NPs in lung injury mouse lungs 2, 4, 8, and 24 hours after particle injection. (**C** and **D**) Blood circulation profile over time in lung injury mice showing the concentration of PEG-based particles remaining in the bloodstream of lung injury mice minutes after particle injection. Plots are shown for both ICAM-1 targeted (T) and untargeted (U) particles. Bars represent the SE for *N* = 4. Statistical analysis was performed using one-way ANOVA with Fisher’s LSD test, where (*) indicates *P* < 0.05 compared to the untargeted particle at that time point.

The targeted, soft MPs (MPST) exhibit statistically increased accumulation in the lung over the untargeted particles (MPSU) at the 2- and 4-hour time points (Fig. 6A), highlighting the benefit of a targeting ligand on the particle surface, which was resolved by the 8-hour time point after particle injection. Figure 6B shows the same comparison for NPS-targeted (NPST) versus untargeted particles (NPSU). There was a statistically increased amount of NPST compared to NPSU at the 2-hour time point after initial particle injection, which was resolved by the 4-hour time point. Notably, the levels of accumulation for the NPST and NPSU were lower than the corresponding values obtained for MPST and MPSU, respectively (fig. S10). Next, we sought to determine whether this difference in adhesion duration between MPs and NPs was due to differences in circulation times by evaluating our hydrogel particles’ pharmacokinetics parameters in vivo with the same LPS model as above. As shown in Fig. 6 (C and D), by the 30-min time point, less than 1% of all particle types were still circulating in the blood. However, the blood concentration for MPST and MPSU dropped the fastest, to less than 0.1% in 30 min, suggesting that the extended presence of the targeted, deformable MPs in the lungs was due to a superior margination and adhesion rather than to a prolonged circulation (see pharmacokinetic parameters in table S1).

## DISCUSSION

It has been reported that less than 1% of intravenously delivered NPs arrive at the targeted tissue or vasculature of interest in cancer applications, which includes both actively targeted and untargeted NPs (*10*, *11*, *28*). This low accumulation of nanocarriers is particularly problematic when carrying cytotoxic agents, e.g., chemotherapy drugs, where precise and highly localized delivery with minimal off-targeted effects are necessary for treatment efficacy. Previous works have suggested that the low nanocarrier accumulation is linked to inefficient margination, where carriers sized 500 nm or less are entrapped within the red blood cell core and thus fail to distribute to the vascular wall in vessels of up to 2 mm size (*13*, *29*). Instead, micrometer-sized particles efficiently distribute to the vascular wall in blood but pose a high risk of occluding the capillaries when constructed from the typical biodegradable polymer.

We previously reported that deformable hydrogel MPs could effectively localize and bind to inflamed vascular wall in vivo in mice with no evidence of occlusion in capillaries (*4*), suggesting an opportunity for their use in drug targeting applications. However, as most vascular-targeted delivery is aimed toward intracellular or tissue application, micrometer-sized particles are inherently challenged for cellular entry or extravasation applications. Here, we engineered our simple emulsion system to achieve hydrogel MPs loaded with polymeric NPs to demonstrate the concept of deformable MPs as transporters for enhancing the delivery of NPs on the vessel wall for vascular-targeted drug delivery applications. While different NP-embedded MP systems have been proposed for theranostic applications (*30*, *31*), none have targeting moieties, nor are they tailored in modulus and NP loading to optimize targeted NP delivery to the vascular wall. We show that targeted hydrogel MPs deliver significantly more NPs to the vessel wall than free, targeted NPs themselves. We confirmed that low adhesion of the freely injected, targeted NPs, which were not PEGylated, was not due to their nonspecific sticking to blood cells or being phagocytosed. Instead, previous works report low NP adhesion to the vascular wall in blood flow due to their inferior margination performance, i.e., NPs colocalized with red blood cells in the core of blood flow (*13*, *15*, *29*, *32*). Our findings shed light on particle modulus as an essential physical property that controls VTCs’ sustained adhesion and the amount of NP cargo transported to the vessel wall (*4*, *20*). Notably, this proposed two-step vascular-targeting strategy can improve the overall targeted delivery efficiency of NPs for broad applications in hard-to-treat diseases.

We also evaluated the critical trade-off between particle circulation time and targeting efficiency, i.e., accumulation in inflamed tissues. For all hydrogel particle types tested, we found extremely short blood circulation times and corresponding half-lives (Fig. 6 and table S1), which was slightly worse for MPs than NPs. We find that the circulation time and resulting half-life of particles is not the most significant indication of particle accumulation to the inflamed vascular wall. The 2-μm hydrogel MPs exhibited superior vascular-targeting efficiency over NPs, as found in our previous in vitro and in vivo works (*12*, *15*, *25*), despite their slightly shorter blood circulation time. Perhaps our most important finding here is that these soft MPs, once adherent, can persist on the vascular wall for hours, which we visualized directly in the mesentery up to an hour (Fig. 5) and indirectly by accumulation in the lung up to 4 to 8 hours (Fig. 6). Thus, these results highlight that rapid margination and retention in the area of interest may be more indicative of a vascular-targeted carrier’s success, which favors micrometer-sized, soft particles (*4*, *13*, *15*), contrary to the long-held notion of the necessity for a long blood circulation time. Given their size of ≥500 nm, we do not expect that hydrogel particles used in this work will be substantially internalized by endothelial cells in the time frame explored (*33*). Therefore, beyond the 4- to 8-hour targeting window, we expect that the endothelial-expressed inflammatory molecules down-regulate as the injury resolves, leading to detachment of bound MPs. On the basis of existing literature, detached particles are expected to mainly redistribute to the liver and spleen, with some fraction sequestered by macrophages (*34*, *35*).

The difference in circulation time between hard and soft particles is not enough to explain the discrepancies in the observed phenomena of relatively rigid hydrogel particle removal after initial binding. We hypothesize that this detachment may occur through multiple mechanisms. For one, leukocytes can potentially disrupt particles by physically colliding with them on the vascular wall and dislodging them (*24*, *36*) or by actively phagocytosing them off the vessel wall (*37*). Rigid particles would be more susceptible to collision and are more readily removed by phagocytic cells (*4*, *36*). In addition, P-selectin’s vascular wall expression is transient by nature, returning to baseline between minutes to hours after the endothelium’s activation, depending on the agonist type (*38*, *39*). A lower P-selectin density expressed by the endothelium in the mesentery will lead to a reduced number of particle receptor-ligand bonds or a weakened adhesion strength, which will negatively affect more rigid particles as they would experience a higher disruptive force exerted by the fluid flow (*40*). Conversely, the soft MPs’ deformability is leveraged to compensate for the weakened adhesion strength; hence, no detachment is observed for these particles throughout the hour (*41*). In addition, no detachment is observed for the PS NPs despite being substantially stiffer than the rigid hydrogels; given their notably smaller size (50 versus 500 nm for the NPHT), they experience a much lower disruptive force from the fluid flow. Overall, these results indicate that MPs reach the vascular wall considerably better than nanometer-sized particles, with softer particles having the maximal potential to maintain any adhesion achieved.

The presented work is limited in that we do not evaluate the release of NPs, which would be a critical component for treatment efficacy. To this end, a degradation mechanism will need to be explored for the hydrogel MP carriers to release the loaded NPs of our proposed system. In this context, researchers have used hydrogel-based materials functionalized for triggered degradation upon exposure to changes in pH (*42*), certain wavelengths of light (*43*), and particular enzymes (*44*). An enzymatic degradation approach is likely the most relevant for vascular-targeted hydrogel MPs because protease activities have been identified in vascular wall cells in diseased tissues, including cancer, inflammation, and cardiovascular diseases (*45*, *46*). For example, the NP cargo can be entrapped, distributed evenly within the hydrogel MP carriers’ matrix that is cross-linked with a protease-susceptible peptide linker (*47*). Upon contact with the vascular wall at the target, MPs are exposed to disease-specific proteases that trigger hydrogel degradation and NP release. Given the up to 4- to 8-hour targeting window identified in this work (Fig. 6A) and numerous works showing that hydrogels can degrade in that time frame (*42*, *43*, *48*), our results suggest that as long as the overall carrier modulus is maintained, there is ample opportunity to tailor the NP release kinetics (*49*). Our future work will explore various possibilities.

In summary, the loading of NPs into hydrogel MPs has excellent potential to improve the delivery of smaller NPs for the many clinical situations that are amenable to targeted drug delivery. Given their highly tunable deformation, these hydrogel carriers can be designed to ensure easy traversal through the vasculature and low risk of vessel occlusion when bound, similar to white blood cells. For all experiments, soft hydrogel MPs offered significantly higher and sustained adhesion than any free NPs, by at least an order of magnitude, demonstrating a massive advantage in trafficking NPs to the vessel wall by loading them into hydrogels. If the same number of NPs was encapsulated into hydrogel MPs, the historically reported value of less than 1% NP accumulation at the target size could be increased by over 10 to 25% of the dose.

## MATERIALS AND METHODS

### Experimental design

Our experiments were designed to investigate deformable hydrogel MPs’ ability to deliver drug-loaded NPs to the vascular wall under in vitro and in vivo blood flow conditions. Particles of different deformability and NP loading ratios were fabricated, and their vessel wall adhesions in flow were quantified and compared to each other both in vitro and in vivo. All the human donors for the experiments were healthy. In vivo experiments were performed on healthy mice. All the experiments were replicated at least three times.

### Study approvals

Human blood used in all assays was obtained via venipuncture according to a protocol approved by the University of Michigan Internal Review Board and in line with the standards set by the Helsinki Declaration of 1975, as revised in 2008. Informed, written consent was obtained from all subjects before blood collection. Phlebotomy was performed according to University of Michigan Medical School Internal Review Board (IRB-MED)–approved protocols and in line with the World Medical Association Declaration of Helsinki. Umbilical cords were obtained under a IRB-MED–approved human tissue transfer protocol (HUM00026898). This protocol is exempt from informed consent per federal exemption category #4 of the 45 CFR 46.101 (b).

Animal studies were conducted following the National Institutes of Health *Guide for the Care and Use of Laboratory Animals* and approved by the Institutional Animal Care and Use Committee of the University of Michigan. C57BL/6 mice were obtained from The Jackson Laboratory. All animals were maintained in pathogen-free facilities at the University of Michigan.

### Rheometry

Swollen shear moduli were measured on an AR-G2 rheometer (TA Instruments), on bulk hydrogels swollen completely in 37°C water, as previously reported (*4*). We fabricated bulk hydrogels with the same composition as the MPs used for the in vitro and in vivo experiments and tested them via rheometry. Briefly, the % final strain was fixed depending on the material properties (0.1% for hard to 10% for soft), and the required strain to achieve the % strain was determined. To convert from shear modulus (*G*) to Young’s modulus (*E*), we use Eq. 1, using a Poisson’s ratio of υ = 0.5, a valid assumption for elastic materials such as swollen hydrogels (*50*)

E=2G(1+υ);Pa (1)

The calculated hydrogel properties in fig. S1A were computed as previously reported (*21*).

### Hydrogel MP fabrication and microscopy

Briefly, PEGDA, 2-carboxyethyl acrylate, acryloxyethyl thiocarbamoyl rhodamine B (rhodamine), and lithium phenyl-2,4,6-trimethylbenzoylphosphinate photoinitiator were combined into methanol at predetermined concentrations, as detailed in fig. S1A. The photoinitiator was synthesized in-house, as previously published (*51*). The fabrication and purification of each hydrogel particle stock were completed, as previously reported (*4*). Briefly, the commercial stock of carboxylated PS NPs (Polysciences) was concentrated via centrifugation and then lyophilized to remove any residual liquid. All other components of the hydrogels were added directly to this dried NP powder to minimize any possible loss by powder transport. The resulting solution was emulsified and polymerized such that the NPs were physically entrapped into the hydrogel matrix. All particle stocks were diluted in 1% polyvinyl alcohol, filtered through a 2-μm filter, and washed at least three times before quantifying the median fluorescence intensity. The volume percent of NPs used in the hydrogel formulation, i.e., 2.3, 3.9, 7.8, and 15.6% (v/v), is reported quantitatively so that NPs of different sizes can be directly compared. To determine the number of NPs loaded per hydrogel MP, a standard curve of NP concentrations was established on a BioTek microplate reader. After thorough washing to remove any free 50-nm NPs, hydrogel MPs were evaluated in a 96-well plate at a fixed concentration of 1 × 10^7^ hydrogel MPs per well. The reading was converted to the number of NPs loaded after subtracting out the unloaded MP background and using the calibration curve. Near-infrared dye was used in place of rhodamine for particle circulation times and biodistribution studies to facilitate detection in whole blood and whole organ scans.

### Particle functionalization and site density

The carboxyethyl acrylate monomer provided terminal carboxylic acid functional handles for all hydrogel conditions. Carboxylated PS particles (Polysciences) were obtained commercially for both loading and free NP studies. All particles were covalently modified with NeutrAvidin Biotin-Binding Protein (Thermo Fisher Scientific), as previously described (*4*), and subsequently incubated either with biotinylated anti-human ICAM-1 (R&D Systems) sites/μm^2^ for flow channel experiments or with anti-mouse CD62P (P-selectin, BD Biosciences) sites/μm^2^ for intravital microscopy to achieve a target site density of 5000 and 30,000 sites/μm^2^, respectively. Surface ligand site densities were quantified as previously described (*36*). Briefly, anti-rat immunoglobulin G2b (IgG2b)–phycoerythrin (PE) and anti-rat IgG1-PE (eBioscience) were used to stain the targeted particles, which were then run on flow cytometry. Standard beads with known fluorescence (Bangs Laboratories) are run along with each particle type to generate a standard calibration curve between median fluorescence intensity and fluorescent surface molecules, which was used to compute the particle antibody site density (# sites/μm^2^) (*32*, *36*). The anti–ICAM-1 and anti–P-selectin site density of particles used in in vitro and in vivo experiments were maintained within 10% of the target # sites/μm^2^.

### Cell culture

HUVECs used in all assays were isolated from healthy umbilical cords, as previously reported (*4*). Briefly, we used a collagenase perfusion method to isolate the HUVECs, which were then maintained in culture for up to four passages. For adhesion assays, the HUVECs are seeded at a confluent cell density onto glass coverslips and used within 24 hours (*13*).

### PPFC adhesion assay

The PPFC assays were conducted in vitro according to our previously published protocol (*4*). Briefly, fresh, anticoagulated blood from healthy adults was mixed with particles of interest and immediately perfused over a HUVEC monolayer activated with interleukin-1β (IL-1β) (Fitzgerald, 1 ng/ml in complete HUVEC medium) for 24 hours in static condition to induce maximal ICAM-1 expression. All particle types were conjugated with 5000 anti-human ICAM-1/μm^2^ to ensure that no trends arose from a difference in targeting ligand densities. For fixed hydrogel concentration experiments, all particle types were mixed at a concentration of 1 × 10^7^ particles/ml of whole blood. For fixed NP concentration experiments, each hydrogel’s concentration was scaled by the number of NPs loaded. The blood and targeted particle mixture were perfused in a laminar flow profile with the WSR (γ_w_) fixed to either 200 or 1000 s^−1^ by control of the volumetric flow rate (*Q*) through the PPFC, calculated as shown in Eq. 2

$$\gamma_{W}=\frac{6Q}{h^{2}w};s^{-1}$$ (2)

where *h* is the channel height (0.0127 cm), *w* is the channel width (0.25 cm), and *Q* is the volumetric flow rate (ml/s). *Q* was 81 and 405 μl/min for the WSRs of 200 and 1000 s^−1^, respectively. After 5 min of blood perfusion, phosphate-buffered saline (PBS) buffer containing 1% bovine serum albumin was added to the PPFC to remove non–firmly adhered particles, and firmly adhered particles were counted and scaled by the monolayer surface area, producing number bound/mm^2^. The translation of hydrogel binding to NPs delivered was performed using a scaling factor, i.e., the average number of NPs loaded per 2-μm hydrogel MP. The numbers were as follows: 15% PEG low loading: 3 NPs per hydrogel MP, 15% PEG high loading: 25 NPs per hydrogel MP, and 50% PEG high loading: 16 NPs per hydrogel MP.

### Flow cytometry analysis of NPs bound to cells

Blood collected after flow assay or after incubation with NPs in static assays was stained with CD11b on ice. After staining for 30 min, leukocytes were fixed using 1× lyse-fix solution and washed three times in PBS before analysis using flow cytometry. A positive shift in the fluorescein isothiocyanate channel compared to nonparticle controls indicated binding of 50-nm PS particles to leukocytes.

### Intravital fluorescent microscopy of murine mesentery vessels

A mouse model of mesenteric inflammation was used to quantify targeted particle adhesion, as described previously (*4*, *25*). Briefly, mice were anesthetized with an intraperitoneal injection of ketamine and xylazine and then opened via an incision along the midline of the abdominal cavity. The mouse’s intestines were manipulated into a U-shape on a heated microscope stage such that the connective tissue laid flat on the slide. A vein (150 to 250 μm in diameter) was then identified by the presence of leukocyte rolling, and TNF-α was topically applied to induce local inflammation. After 3 min, we injected the particle dose of interest in a total volume of 200 μl. After 5 min, firm adhesion was quantified and scaled per area of the blood vessel, resulting in #/mm^2^ vessel. For these experiments, all particles were conjugated with 30,000 anti-mouse P-selectin sites/μm^2^ to ensure that no trends observed were due to differences in surface ligand density. The NPs delivered were again calculated by scaling by the number of NPs loaded per hydrogel MP.

To determine how long particles stay bound, intravital microscopy was conducted as previously described (*4*, *25*) but modified to extend the time window. A vein was visualized, and topical TNF-α was dropped topically in the area of interest. After 3 min, particles (30 mg/kg) were injected via a tail vein catheter. Firm particle adhesion was quantified every 5 min by taking five pictures in the same physical locations and scaled per area of the blood vessel, resulting in #/mm^2^ vessel. About 50 μl of the ketamine and xylazine mixture was dropped topically onto the exposed abdominal cavity every 20 min to maintain the anesthesia of the mice.

### Circulation time and pharmacokinetic parameters of PEG particles

Circulation time curves of particles were established via a tail nick procedure. Particles were prepped with 10,000 anti–ICAM-1/μm^2^ and dosed by equivalent mass (~30 mg/kg) by injection through a tail vein catheter. At each time point after tail vein injection (5 min, 30 min, 1 hour, 3 hours, and 24 hours), 10 μl of blood was collected and diluted 10× into PBS. The blood samples were deposited into black-sided 96-well plates and scanned on the IVIS. The signal intensity was converted to particle concentration by a standard curve of each particle type. These values were converted to mass circulating by multiplying by the mass of a single particle.

Pharmacokinetic analysis of the circulation time data was completed in PKSolver. Both one- and two-compartment models were tested for all conditions; two-compartment models were more accurate for all conditions. The biexponential fit parameters are summarized in table S1.

### Whole-organ near-infrared scanning

Whole-organ scans were performed on an Odyssey CLx Infrared Imaging System (LI-COR) using the 700-nm channel at 169-μm resolution. Total fluorescence for each organ was determined by drawing a region of interest (ROI) using Image Studio Software (LI-COR). Untreated samples were used to determine each organ’s background fluorescence, which was subtracted from the fluorescence obtained for each organ ROI.

### Quantification of particle mass per organ

To account for variability in particle brightness, standard curves of each particle type were generated using the Odyssey LI-COR Imaging System. Total fluorescence values of each organ were converted to the number of particles per organ using these standard curves. For each mouse, this value was then scaled by each organ’s weight to achieve a mass of particles per mass of organ. Total recovered fluorescence was determined as the sum from each organ. The corresponding fluorescent percentage was determined as a portion of this total.

### Statistics

Rheometry data represent three different bulk hydrogels each, with at least 15 data points from each hydrogel. The loading of NPs was measured for two different batches of hydrogels fabricated. The parallel plate flow experiment data are an average of 10 pictures from each experiment, with *n* ≥ 3 blood donors for each group of data presented. Intravital results represent at least three mice per group, with at least 10 images of particle adhesion quantified from each mouse. All mesentery data represent the average of three mice. All circulation time data represent the average of four mice each. All data were included for all figures presented in this paper, and no outliers were excluded for any reason. In all figures, the error bars represent the SEM. As indicated in figure legends, the asterisks denote statistical *P* values between the designated bars, with (*) indicating *P* < 0.05, (**) indicating *P* < 0.01, (***) indicating *P* < 0.001, and (****) indicating *P* < 0.0001. A lack of asterisks denotes that the difference is not significant.

## SUPPLEMENTARY MATERIALS

Supplementary material for this article is available at http://advances.sciencemag.org/cgi/content/full/7/17/eabe0143/DC1

## Appendix

View/request a protocol for this paper from *Bio-protocol*.
